# Hydrogen Generation from Al-NiCl_2_/NaBH_4_ Mixture Affected by Lanthanum Metal

**DOI:** 10.1100/2012/150973

**Published:** 2012-04-24

**Authors:** Wen Qiang Sun, Mei-Qiang Fan, Yong Fei, Hua Pan, Liang Liang Wang, Jun Yao

**Affiliations:** College of Materials Science and Engineering, China Jiliang University, Hangzhou 310018, China

## Abstract

The effect of La on Al/NaBH_4_ hydrolysis was elaborated in the present paper. Hydrogen generation amount increases but hydrogen generation rate decreases with La content increasing. There is an optimized composition that Al-15 wt% La-5 wt% NiCl_2_/NaBH_4_ mixture (Al-15 wt% La-5 wt% NiCl_2_/NaBH_4_ weight ratio, 1 : 3) has 126 mL g^−1 ^min^−1^ maximum hydrogen generation rate and 1764 mL g^−1^ hydrogen generation amount within 60 min. The efficiency is 88%. Combined with NiCl_2_, La has great effect on NaBH_4_ hydrolysis but has little effect on Al hydrolysis. Increasing La content is helpful to decrease the particle size of Al-La-NiCl_2_ in the milling process, which induces that the hydrolysis byproduct Ni_2_B is highly distributed into Al(OH)_3_ and the catalytic reactivity of Ni_2_B/Al(OH)_3_ is increased therefore. But hydrolysis byproduct La(OH)_3_ deposits on Al surface and leads to some side effect. The Al-La-NiCl_2_/NaBH_4_ mixture has good stability in low temperature and its hydrolytic performance can be improved with increasing global temperature. Therefore, the mixture has good safety and can be applied as on board hydrogen generation material.

## 1. Introduction

Hydrogen is a nonpolluting fuel and a clean energy which can be consumed and transformed to electricity in proton exchange membrane fuel cell (PEMFC). The development of PEMFC in vehicles and portable electronics requires large amounts of hydrogen storage at moderate temperature with high efficiency and low cost [1]. On board hydrogen generation from reaction of chemical hydrides or metals in aqueous solutions can storage more hydrogen at moderate temperature in comparison to conventional hydrogen storage materials [2]. Among these hydrogen generation materials, sodium borohydride (NaBH_4_) and aluminum (Al) have been paid attention widely due to high theoretic hydrogen generation density, safe, controllable, and mild operating conditions. Many achievements showed that NaBH_4_- and Al-based hydrogen generation system attached to PEMFC can provide 2 W–10 kW powers [3, 4]. Nevertheless, NaBH_4_ and Al have many disadvantages. For example, NaBH_4_ is an expensive raw material and alumina layer on Al surface reduces Al reactivity in water. The low solubility of NaBH_4_ and NaBO_2_ reduce hydrogen generation density and catalyst reactivity and durability [5]. So the disadvantages limit the commercial application of these hydrogen generation materials.

Hydrogen generation from solid-stage NaBH_4_ or NaBH_4_/Al mixture in little water amount presents a good solution to overcome the disadvantages of traditional NaBH_4_- and Al-based hydrogen generation system. It has high hydrogen generation density and fast hydrolytic kinetic as exothermal reaction proceeds in limited water amount. Liu et al. [6] found that the uniform mixture of solid-state NaBH_4_ and Ru-based catalyst has 7.3 wt% hydrogen generation density at 298 K, meeting the required target of USA department of energy (6.5 wt.%). Sodium borohydride/nanoaluminum could supply stable hydrogen generation with about 7 wt.% yield when their mass ratio is 1 : 1 [7]. Dai et al. [8] found that micro-Al/NaBH_4_/NaOH mixture had controllable hydrogen generation performance via regulating the amount and rate of CoCl_2_ solution. In such case, water acts as an oxidizer for both aluminum and metal borohydride, thus as a source of hydrogen. There also exists an interaction of Al/NaBH_4_ hydrolysis which improves their hydrolytic kinetics. However, there is a great shortcoming that nanoaluminum particle or alkaline solution has to be used to start the hydrolytic reaction. It is necessary to find an environmental-friendly metal which can improve aluminum reactivity in neutral aqueous solution at moderate temperature. Metal lanthanum is a good candidate. It is a good hydrogen generation material and its hydrolysis byproduct presents alkaline, which accelerates the hydrolysis kinetic of magnesium and aluminum [9, 10]. Luo et al. [11] found that the addition of rare earth metal into aluminum alloy could effectively damage the dense oxide layer and led to the formation of flower-like nanoflake reaction products, which prevented the byproducts Al(OH)_3_ from depositing densely on the surface of composites and stimulated Al hydrolysis.

We herein report a simple but effective method that can control hydrogen generation performance of micro-Al-NiCl_2_/NaBH_4_ mixture and reduce the alkali corrosion problems via the lanthanum additive (La). The relative hydrolysis mechanism has been discussed to gain the mechanistic understanding of the effect of La.

## 2. Experimental

### 2.1. Preparation of Al-La-NiCl_2_ Mixture

Elemental aluminum powder (99.9% purity and particle size of about 10 *μ*m; Angang Group Aluminum Powder Co., Ltd., China), La powder (99.0% purity), NiCl_2_ (99.0% purity), and NaBH_4_ (98% purity; China Chemical Company, Ltd.) were used as starting materials. The composites of the Al-15 wt.% La-5 wt.% NiCl_2_ mixture (if not specially noted) were weighed and mixed in an argon-filled glove box. The total weight of the mixture was 2 g, and ball milling was performed by a QM-ISP3 planetary ball miller under 0.2–0.3 MPa argon atmosphere. Ball-to-powder mass ratio corresponded to 30 : 1 at a milling time of 15 h and a rotation speed of 400 r/min.

### 2.2. Hydrolytic Performances and Microstructure of Al-La-NiCl_2_/NaBH_4_ Mixture

Hydrolytic performances of the Al-La-NiCl_2_/NaBH_4_ mixture with different weight ratios were carried out in pure water at 323 K. The total weight of the Al-La-NiCl_2_/NaBH_4_ was 0.4 g, while the volume of pure water was 100 mL. Weight ratio of the Al-La-NiCl_2_/NaBH_4_ was 1 : 1, unless otherwise stated. At the set temperature, the mixture was placed in water and the produced gas flowed through a condenser prior to measurement of the hydrogen volume. The produced hydrogen volume was measured by monitoring the water displaced from a graduated cylinder at 273 K and 1 atm as the reaction proceeded, in accordance with the experiment conducted by Soler et al. [12]. The reaction time began with the first bubble, and the final volume of the produced hydrogen was collected after 60 min of reaction. Conversion efficiency was calculated according to the following equation: % = experimental hydrogen generation amount/theoretical hydrogen generation amount × 100%. Impurities were involved in the calculations. Powder X-ray diffraction (XRD) studies were carried out in an X-ray diffractometer (RIGAKU, Japan, model D/MAX2550V/PC).

## 3. Results and Discussion

**Figure 1:**
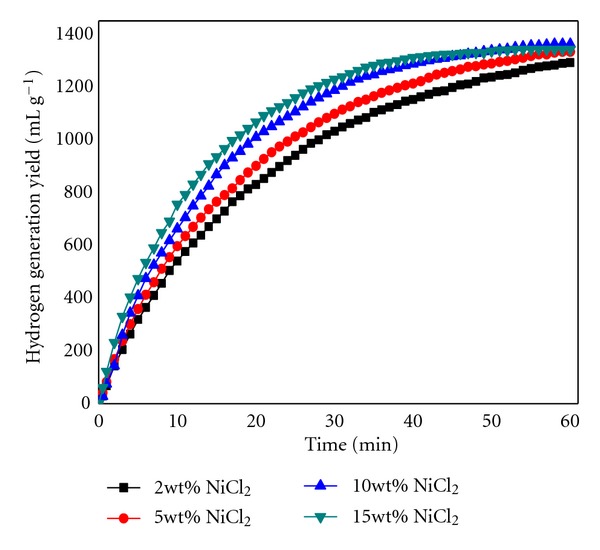
Hydrogen generation performance of Al-15 wt. %La-*x* wt. %NiCl_2_/NaBH_4_ mixture (weight ratio, 1 : 1) at 323 K. *x*: 2, 5, 10, 15.

NiCl_2_ is a good promoter for NaBH_4_ hydrolysis. In the present study, we firstly examined effect of NiCl_2_ on hydrogen generation performance of Al/NaBH_4_ hydrolysis. As seen in Figure 1, increasing NiCl_2_ content results in an increasingly favorable hydrogen generation rate and amount of Al/NaBH_4_ mixture. The Al-15 wt. %La-2 wt.% NiCl_2_/NaBH_4_ (weight ratio, 1 : 1) mixture shows 68 mL g^−1 ^min^−1^maximum hydrogen generation rate and yields 1293 mL g^−1^within 60 min. Upon increasing NiCl_2_ content to 15 wt%, hydrogen generation maximum rate and amount of the mixture include 122 mL g^−1 ^min^−1^ and 1362 mL g^−1^, respectively. Reaction (1) of NiCl_2_ and NaBH_4_ generates Ni_2_B in the hydrolysis process. Ni_2_B has high catalytic reactivity on NaBH_4_ hydrolysis, especially distributed into Al(OH)_3_ particle [13].

(1) $$2\text{Ni}{\text{Cl}}_{2}+4\text{Na}{\text{BH}}_{4}+6{\text{H}}_{2}\text{O}\to {\text{Ni}}_{2}\text{B}+3\text{H}{\text{BO}}_{2}+12.5{\text{H}}_{2}.$$

Meanwhile, Ni_2_B deposits on Al surface and acts as a cathode of a microgalvanic cell (Al-Ni_2_B), which stimulates the electrochemical corrosion of Al according to (2). Therefore, higher NiCl_2_ content leads to more Ni_2_B amount, which accelerates Al/NaBH_4_ hydrolysis correspondingly.

(2) $$\begin{array}{c} \text{At}\;\text{the}\;\text{anode:}\;\text{Al}+3{\text{H}}_{2}\text{O}-3\text{e}\to \text{Al}{\left(\text{OH}\right)}_{3}+3{\text{H}}^{+}.\\ \text{At}\;\text{the}\;\text{cathode:}\;3{\text{H}}^{+}\left({\text{Ni}}_{2}\text{B}\right)+3\text{e}\to 1.5{\text{H}}_{2}. \end{array}$$

**Figure 2:**
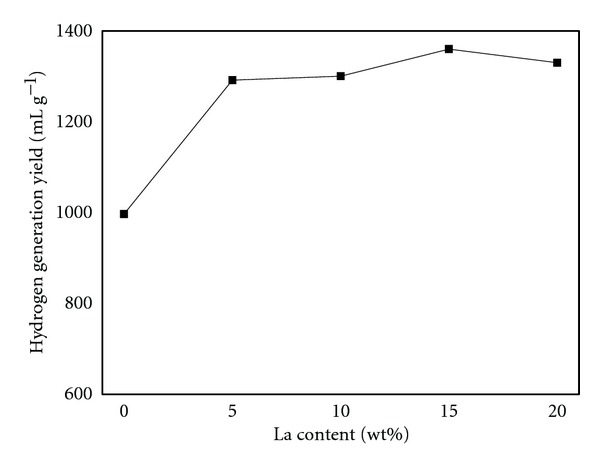
Relationship of hydrogen generation amount and La content at 323 K.

**Figure 3:**
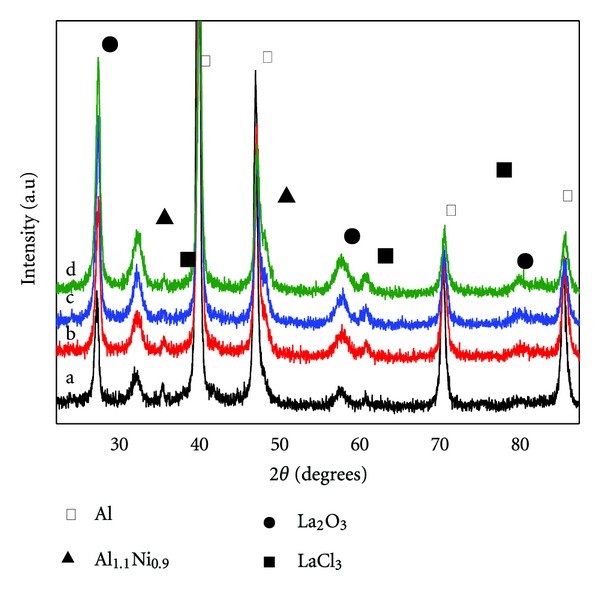
XRD results of Al-*x* wt% La-5 wt% CoCl_2_ mixture. *x*: a, 5; b, 10; c, 15; d, 20.

The addition of a small amount of La metal causes a remarkable increase of hydrogen generation performance of Al/NaBH_4_ mixture. The observation provides new possibilities for practical composition designs. In our preliminary study of Al-La-NiCl_2_/NaBH_4_ mixture, we develop sets of control experiments to optimize the composition design. For comparison purposes, the composition of Al-*x* wt% La-5 wt% NiCl_2_/NaBH_4_ (weight ratio, 1 : 1) was fixed except Al and La contents. The La content was fixed at 0, 5, 10, 15, and 20 wt%. We examined the impact of La content on hydrogen generation performance of the mixture. The results are shown in Figure 2. The hydrogen generation amount increase with increasing La content and reach maximum values of 1360 mL g^−1^ within 60 min at 15 wt% La content. The efficiency is 82%. Further increasing La content deteriorates hydrogen generation performance. It can be explained from the microstructure change in Figure 3, which shows XRD results of Al-5 wt% NiCl_2_ with different La content. The peaks of La, Al, and AlNi alloy are identified, reflected that La and Al have not formed alloy compound in the milling process. However, broadened lines are observed with increasing La content. Combined with the Scherrer equation (*D*_hkl_ = *kλ*/*β*cos⁡*θ*_hkl_), the crystal size can be roughly calculated. Its value decreases from 238 Å to 190 Å with La content increasing from 5 to 15 wt%, but further increases to 197 Å with La content further increasing to 20 wt%. In addition, La metal has high reactivity and reacts with water to produce some hydrogen at 298 K.

**Figure 4:**
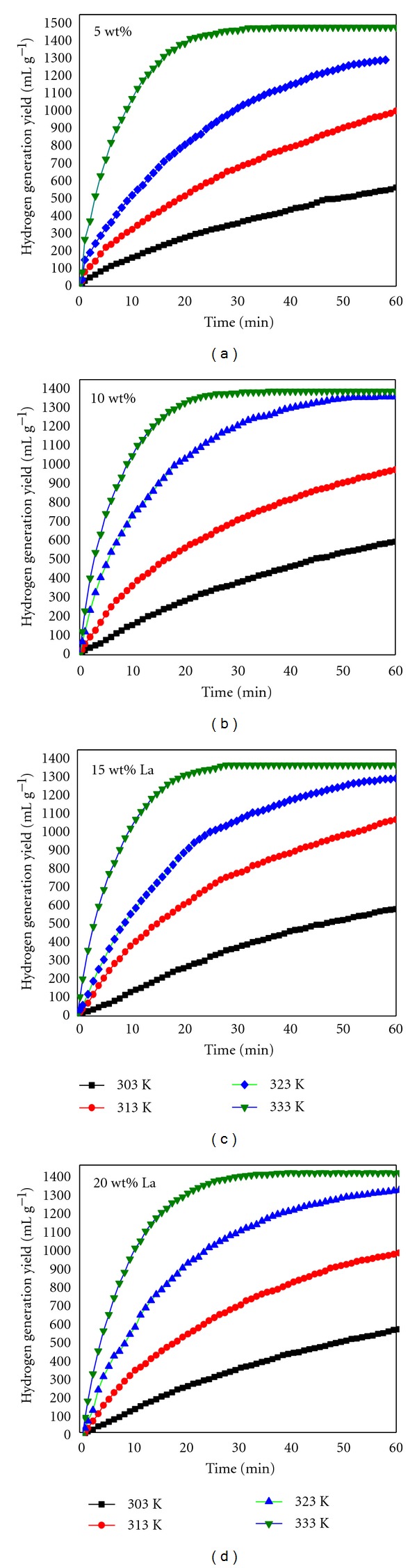
Hydrogen generation performance of Al-*x* wt.% La-5 wt.% NiCl_2_/NaBH_4_ mixture (weight ratio, 1 : 1) at different temperatures. *x*: 5; 10; 15; 20.

However, the hydrogen generation rate is conversely proportional to La content, which can be obviously obtained in Figure 4. At same set temperature, the maximum hydrogen generation rate decreases from with La content increasing. For example, the value of maximum hydrogen generation rate decreases from 267 to 230, 201, and 187 mL g^−1 ^min^−1^ with La content increasing from 5 to 10, 15, and 20 wt%, respectively. It is known that hydrolysis byproduct La(OH)_3_ has low solubility in water and deposits on Al surface. So the contact area of Al and H_2_O is decreased and hydrogen generation rate is reduced therefore. 

Al-La-NiCl_2_/NaBH_4_ mixture has low reactivity in low temperature. The results in Figure 4 show that the mixture only yields approximate 500 mL g^−1^ with about 30% efficiency at 303 K. In practical application, the mixture seldom reacts with water and has good stability at 298 K. But with increasing global temperature, hydrogen generation performance can be improved. In order to further understand the effect of La on hydrogen generation performance of Al/NaBH_4_ mixture, the relationship of activation energy and La content was analyzed. Using the Arrhenius equation (3):

(3) $$k=A\exp\left(-\frac{Ea}{RT}\right).$$

**Figure 5:**
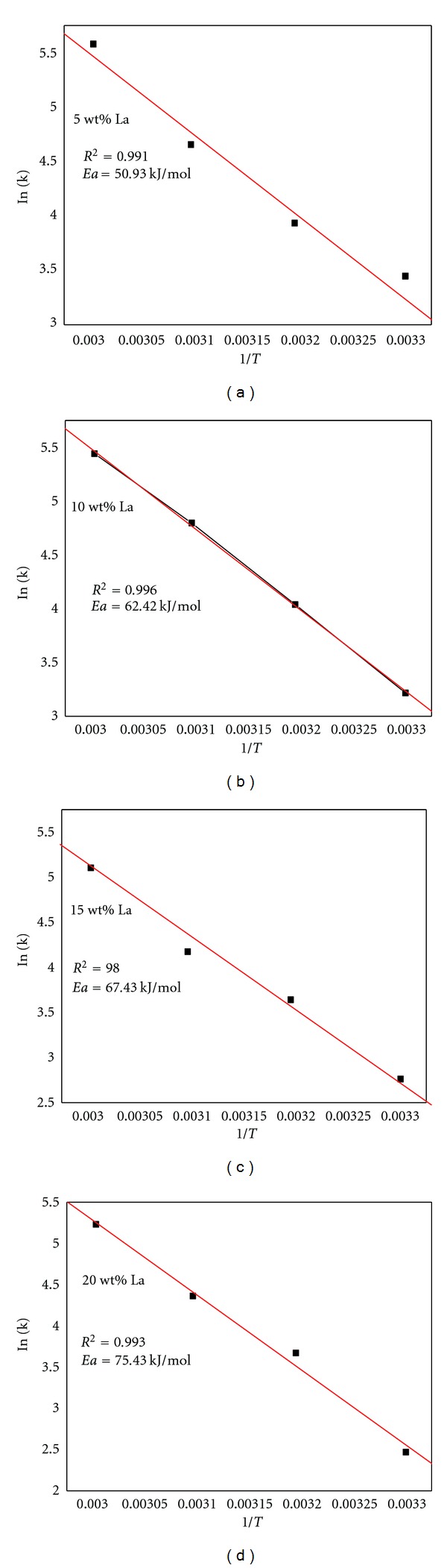
Arrhenius plot of the rate constants using Al-*x* wt% La-5 wt% NiCl_2_/NaBH_4_ (Al-La-NiCl_2_/NaBH_4_ weight ratio, 1 : 1). *x*: 5, 10, 15 and 20 wt%.

**Figure 6:**
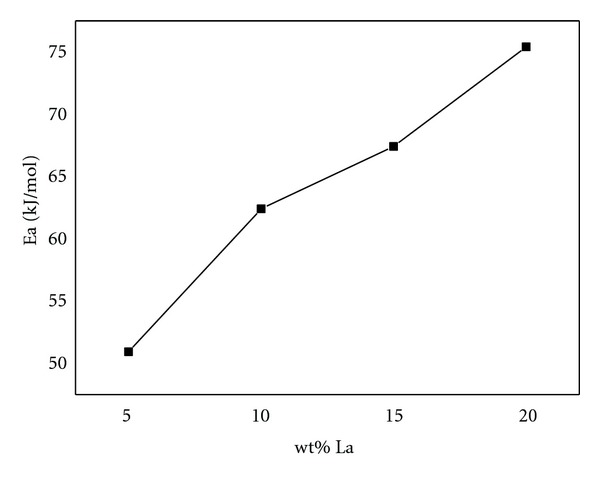
Relationship of activation energy (*Ea*) and La content.

The equation (2) gives the dependence of a rate constant (*k*) on the temperature (*T*) and the activation energy (*Ea*). The values of *k* can be determined from the maximum hydrogen generation rate at different temperatures. Using the linear relationship of In⁡*k* ~ 1/*T* in Figure 5, the activation energy of Al/NaBH_4_ mixture with different La content can be calculated. It can be found that activation energy is increased with increasing La content in Figure 6. The activation energy values are 50.9, 62.4, 67.5, and 75.2 kJ/mol when the La content are corresponded to 5, 10, 15, and 20 wt % of Table 1 shows hydrogen generation amount and maximum rate of Al-15 wt% La-5 wt% NiCl_2_/NaBH_4_ with different weight ratios. Al-15 wt% La-5 wt% NiCl_2_ has low hydrogen generation rate and amount, but the hydrogen generation performance is increased with decreasing weight ratios of Al-15 wt.% La-5 wt.% NiCl_2_/NaBH_4_, especially that Al-15 wt.% La-5 wt.% NiCl_2_/NaBH_4_ with weight ratio of 1 : 3 has best hydrogen generation performance. Its maximum hydrogen generation rate and amount are up to 126 mL g^−1 ^min^−1^ and 1764 mL g^−1^ within 60 min. The efficiency reaches 88%. La and NiCl_2_ have little effect on Al hydrolysis, but seriously affect NaBH_4_ hydrolysis. The hydrolysis byproducts of NaBH_4_ hydrolysis stimulate Al hydrolysis in some degree, according to reactions (2).

## 4. Conclusions

La additive has greatly affects hydrogen generation performance of Al/NaBH_4_ mixture. Hydrogen generation amount increases but hydrogen generation rate decreases with La content increasing. There is an optimized composition that Al-15 wt% La-5 wt% NiCl_2_/NaBH_4_ mixture (weight ratio, 1 : 3) has 126 mL g^−1 ^min^−1^ maximum hydrogen generation rate and 1764 mL g^−1^ hydrogen generation amount within 60 min. The addition of La has great effect on NaBH_4 _hydrolysis but little effect on Al hydrolysis at 323 K. Increasing La content is helpful to decrease the particle size of A-La-NiCl_2_ in the milling process, which induces that the hydrolysis byproduct Ni_2_B is highly distributed into Al(OH)_3_ and the catalytic reactivity of Ni_2_B/Al(OH)_3_ is increased therefore. Meanwhile, there exists a synergistic effect of hydrolysis byproduct NaBO_2_ and Ni_2_B to simulate Al hydrolysis. The Al-La-NiCl_2_/NaBH_4_ mixture has good stability in low temperature and its hydrolytic performance can be improved with increasing global temperature. So the mixture has good safety and can be applied as on board hydrogen generation material.

## Figures and Tables

**Table 1:** Hydrogen generation amount and maximum rate of Al-15 wt% La-5wt% NiCl_2_/NaBH_4_ with different weight ratios.

Al-15 wt% La-5wt% NiCl_2_/NaBH_4_ weight ratio	Maximum hydrogen generation (mL g^−1^ min^−1^)	Hydrogen generation Amount (mL g^−1^)	Efficiency (%)
0 : 4	115	1511	58
1 : 3	126	1764	88
1 : 1	58	1294	78
3 : 1	54	700	52%
4 : 0	15.4	60	6%
